# Nr2e1 Deficiency Augments Palmitate-Induced Oxidative Stress in Beta Cells

**DOI:** 10.1155/2016/9648769

**Published:** 2015-11-16

**Authors:** Xiaoli Shi, Haohua Deng, Zhe Dai, Yancheng Xu, Xiaokan Xiong, Pei Ma, Jing Cheng

**Affiliations:** Department of Endocrinology, Zhongnan Hospital of Wuhan University, Wuhan, Hubei 430071, China

## Abstract

Nuclear receptor subfamily 2 group E member 1 (Nr2e1) has been regarded as an essential regulator of the growth of neural stem cells. However, its function elsewhere is unknown. In the present study, we generated Nr2e1 knockdown MIN6 cells and studied whether Nr2e1 knockdown affected basal beta cell functions such as proliferation, cell death, and insulin secretion. We showed that knockdown of Nr2e1 in MIN6 cells resulted in increased sensitivity to lipotoxicity, decreased proliferation, a partial G0/G1 cell-cycle arrest, and higher rates of apoptosis. Moreover, Nr2e1 deficiency exaggerates palmitate-induced impairment in insulin secretion. At the molecular level, Nr2e1 deficiency augments palmitate-induced oxidative stress. Nr2e1 deficiency also resulted in decreases in antioxidant enzymes and expression level of Nrf2. Together, this study indicated a potential protective effect of Nr2e1 on beta cells, which may serve as a target for the development of novel therapies for diabetes.

## 1. Introduction

In type 1 diabetes (T1D) beta cell mass is markedly reduced by autoimmune destruction. Type 2 diabetes (T2D) results from inadequate beta cell mass and function that can no longer compensate for insulin resistance. While type 1 diabetes clearly results from a loss of beta cells, the contribution of beta cell failure to type 2 diabetes is uncertain for decades [1]. It is increasingly being realized that T2D only develops in insulin resistant subjects with beta cell dysfunction [2, 3]. In insulin-resistant states, pancreatic islets usually respond by beta cell compensation, a process which involves both expansion of the beta cell mass and enhanced beta cell function to maintain normoglycemia [4–6]. Defects of this beta cell compensatory response trigger the onset of diabetic hyperglycemia. Thus, a more complete understanding of the mechanisms that control islet beta cell survival and function should provide new clues to develop more effective therapies for both major forms of diabetes.

Nuclear receptor subfamily 2 group E member 1 (NR2E1) is a member of nuclear receptor superfamily [7]. Studies of gene knockout mice indicate that Nr2e1 plays a significant role in the development of nervous systems [8]. Loss of Nr2e1 function in mouse leads to reduced brain size, visual impairment, and also lower total body weight and less fat deposition [9]. Further functional analyses reveal that Nr2e1 plays important roles in maintaining the proliferative state of adult neural stem cells [10] and retinal progenitor cells [11] via its direct transcriptional control of some cell-cycle regulators and regulation of Wnt/*β*-catenin, MAPK (mitogen-activated protein kinases), and other signaling pathways [12–14]. Nr2e1 also is involved in regulation of Ca^2+^ release. It has been reported that expression of Plce1 (a member of the PLC family which leads to Ca^2+^ release), Cacna1d, and Cacng5 (two L type, voltage-gated calcium channels) was notably upregulated in the Nr2e1−/− retinas [11]. In neural stem cells, it has been observed that TLX negatively regulates CDKN1A expression [15]. A recent study demonstrated that Nr2e1 also performs a positive role in prostate cancer growth regulation via its transcriptional coregulation of CDKN1A and SIRT1 genes [16]. Since (1) CDKN1A and SIRT1 also play important roles in regulating beta cells function and (2) beta cells function is associated with intracellular Ca^2+^ which can be regulated by Nr2e1, it is possible that Nr2e1 may also control the function of beta cells.

In the current study, using MIN6, a mouse insulinoma cell line which exhibits the ability of glucose stimulated insulin secretion similar to the isolated pancreatic islet [17] and has been widely used as beta cell specific cell line [18], we addressed possible functions of Nr2e1 which may be of interest to the field of diabetes.

## 2. Materials and Methods

### 2.1. Cell Culture

Murine MIN6 cells were maintained in DMEM (HyClone) containing 25 mM glucose supplemented with 15% (vol/vol) FCS (Gibco), 2 mM L-glutamine, streptomycin (0.1 mg/mL), and benzylpenicillin (100 U/mL) at 37°C in a humidified 5% CO_2_ atmosphere. Culture media were changed every 2 to 3 days of culture.

### 2.2. Generation of Nr2e1–shRNA MIN6 Cell Lines

Short-hairpin sequences against the Nr2e1 gene or the scrambled shRNA sequences were cloned into the GFP-labeled lentiviral vector GV115 (GENECHEM). The target sequences selected are as follows: Nr2e1–sh1, 5′-CCGATTACAAGACTACTTT-3′; Nr2e1–sh2, 5′-AGAAGCTGAACAAGATCAT-3′; Nr2e1–sh3, 5′- GGTTGATGCTAACACTCTA -3′; and scrambled shRNA(shc), 5′- TTCTCCGAACGTGTCACGT -3′. MIN6 cells were transduced with the shRNA lentiviral particles (100 multiplicity of infection), and GFP-negative and GFP-positive cells were sorted by fluorescence-activated cell sorting (FACS) using the FACS Calibur flow cytometer instrument (BD Biosciences). Nr2e1 mRNA expression levels were determined by RT-PCR, and protein expression was confirmed by immunoblotting.

### 2.3. Real-Time PCR

Total RNA was isolated from cultured MIN6 cells using TRIzol (Invitrogen) and reverse transcribed using M-MLV reverse transcriptase (Promega). Quantitative RT-PCR analysis was carried out using the SYBR Green Taq ReadyMix (Takara) on a Light Cycler 2.0 instrument (Roche); diluted cDNA was used as template. ACTB was used as endogenous control. Relative gene expression level was calculated using the comparative Ct method formula 2^−ΔΔCt^. The sequences of primers for PCR were listed in Supplemental Table 1 in Supplementary Material available online at http://dx.doi.org/10.1155/2016/9648769.

### 2.4. Immunoblot Analysis

MIN6 cells were cultured in 6-well plates. The cells were washed in cold PBS and lysed in protein lysis buffer. Protein was separated on SDS-PAGE gel, electroblotted onto a PVDF membrane, and incubated with 5% BSA in TBS with 0.1% Tween 20, and the membranes were probed with the primary antibodies anti-Nr2e1 (Santa Cruz, 1 : 500), anti-Nrf2 (Santa Cruz, 1 : 200), and anti-GAPDH (Epitomics, 1 : 10000). Horseradish peroxidase-conjugated anti-rabbit immunoglobulin G was used as secondary antibody at a concentration of 1 : 10000 (KPL). Signals were detected using a chemiluminescence assay system (Millipore).

### 2.5. MTT Assay

Cell viability was determined by the 3-[4, 5-dimethylthiazol-2-yl]-2,5-dephenyl tetrazolium bromide (MTT) (Sigma) following the manufacturer's protocol. MIN6-shc, MIN6-sh2, and MIN6-sh3 cells were seeded in 96-well microtitre plates with 5 × 10^3^ per well and incubated in complete medium with 0.5 mM palmitic acid for 48 hours. At the end of the exposure, 20 *μ*L of MTT solution (5 mg/mL) was added to each well, and cells were additionally incubated at 37°C for 4 hours. After the supernatant fluid was removed, 150 *µ*L of solubilization solution (DMSO) was added to each well and shaken for 10 minutes. Absorbance was measured at 570 nm, with a microplate reader.

### 2.6. EdU Incorporation Assay

EdU incorporation assay was carried out using the Cell-Light TM EdU imaging detecting kit according to the manufacturer's instructions (RiboBio). Briefly, MIN6-shc, MIN6-sh2, and MIN6-sh3 cells (1 × 10^4^) were cultured in 96-well plates for 24 h and exposed to 50 *μ*M EdU for 4 h at 37°C. The cells were then fixed in 4% paraformaldehyde for 30 min at room temperature and permeabilized in 0.5% Triton X-100 for 10 min. Cells were washed with PBS and then incubated with staining solution for 30 min. Then the cells were stained with Hoechst 33342 (200 *μ*L per well) for 30 min and imaged under a fluorescent microscope.

### 2.7. Cell-Cycle Analysis

MIN6-shc, MIN6-sh2, and MIN6-sh3 cells were trypsinized, washed with cold PBS, and fixed in ice cold 70% (vol/vol) ethanol. Before analysis, cells were subsequently washed twice with PBS, incubated for 30 min at room temperature with RNase A (100 *μ*g/mL), and stained with PI (2.5 *μ*g/mL) for 30 min. Then cells were analyzed on a FACScan (BD Biosciences).

### 2.8. Determination of Cell Apoptosis

The level of cell apoptosis was measured by 7-AAD and PE staining. MIN6-shc, MIN6-sh2, and MIN6-sh3 cells were exposed to 0.5 mM palmitic acid overnight. Cells were vital-stained with PE and 7-AAD and examined by a flow cytometry (BD Biosciences). Cells which are negative for both dyes are viable cells. The cells positive only for PE were early apoptotic and those which were positive only for 7-AAD were necrotic. The cells positive for both dyes were considered late apoptotic cells. The early and late apoptotic cells were combined and represented as total apoptosis. The apoptosis index was calculated as the percentage of early and late apoptotic cells divided by the total number of cells.

### 2.9. Insulin Secretion Assay

Insulin secretion was measured in static conditions at 37°C as previously described [19]. After a 30 min starvation in DMEM without glucose, insulin secretion was measured during 2 h in HEPES-balanced Krebs-Ringer bicarbonate buffer (125 mM NaCl; 5.9 mM KCl; 1.2 mM MgCl_2_; 1.3 mM CaCl_2_; 25 mM HEPES, pH 7.4) containing 0.1% BSA and different glucose concentrations (2.5 mM or 20 mM). At the end of incubation, insulin concentration was measured in the medium using a mouse insulin Elisa kit (Cusabio) and normalized by cell protein content. To test insulin secretion in lipotoxic conditions, MIN6-shc, MIN6-sh2, and MIN6-sh3 cells were cultured for 48 h in DMEM with 0.5 mM palmitate. Insulin secretion was then measured in static conditions as described above.

### 2.10. Measurement of Reactive Oxygen Species (ROS) Production

The generation of ROS was measured using an oxidation-sensitive fluorescent probe dihydroethidium (DHE) (Beyotime). MIN6-shc, MIN6-sh2, and MIN6-sh3 cells were exposed to 0.5 mM palmitic acid for 24 h. Cells were then incubated with 5 *μ*M of DHE for 30 min at 37°C and then analyzed by flow cytometry. Mean fluorescence intensity was obtained.

### 2.11. Measurement of SOD, GSH-Px, and GSH

The defense systems against free radical attack were assessed by the measurement of both the activities of superoxide dismutase (SOD) and glutathione peroxidase (GSH-Px), using the corresponding commercial kit from Nanjing Jiancheng Bioengineering Institute (Jiancheng). Glutathione (GSH) levels were quantified using a commercial reduced GSH assay kit (Jiancheng), and the amount of GSH was expressed in terms of milligram of GSH per gram of proteins.

### 2.12. Statistical Analysis

Results shown are the mean ± SD. Statistical significance between two experimental conditions was analyzed by using Student's *t*-test. The *P* < 0.05 was considered statistically significant.

## 3. Results

### 3.1. Generation of Nr2e1 Knockdown MIN6 Cells

**Figure 1:**
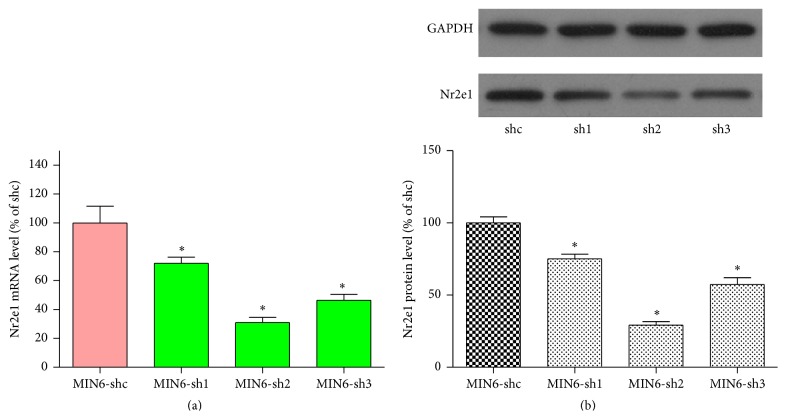
Knockdown of Nr2e1 results in reduced expression of Nr2e1 in MIN6 cells. (a) mRNA expression of Nr2e1 in MIN6 cells transduced with shRNA lentivirus targeted against mouse Nr2e1 (MIN6-sh1, MIN6-sh2, and MIN6-sh3) or scrambled nontarget negative control (MIN6-shc). (b) Reduced protein expression of Nr2e1 MIN6-sh1, MIN6-sh2, and MIN6-sh3 cells. Results are means ± SD for three observations. ^*∗*^ *P* < 0.05. All comparisons with MIN6-shc cells.

We downregulated Nr2e1 protein levels by transduction with lentiviral vectors expressing shRNA that specifically targets Nr2e1 mRNA. Three shRNA sequences targeted to different sites of Nr2e1 mRNA were used, and the transduced MIN6 cells were named MIN6-sh1, MIN6-sh2, and MIN6-sh3. A lentiviral vector carrying a scrambled shRNA sequence was used to create transduced control cells (MIN6-shc). Downregulation of Nr2e1 was confirmed by RT-PCR showing reductions of Nr2e1 mRNA levels by 28% (sh1), 69% (sh2), and 54% (sh3) (Figure 1(a)). A further confirmation was obtained by the western blot analyses showing a decrease of Nr2e1 protein levels by 25%, 71%, and 43%, respectively (Figure 1(b)). Since the sh2 and sh3 target sequences are more effective, they were used for later experiments.

### 3.2. Nr2e1 Knockdown MIN6 Cells Exhibit Increased Sensitivity to Lipotoxicity

**Figure 2:**
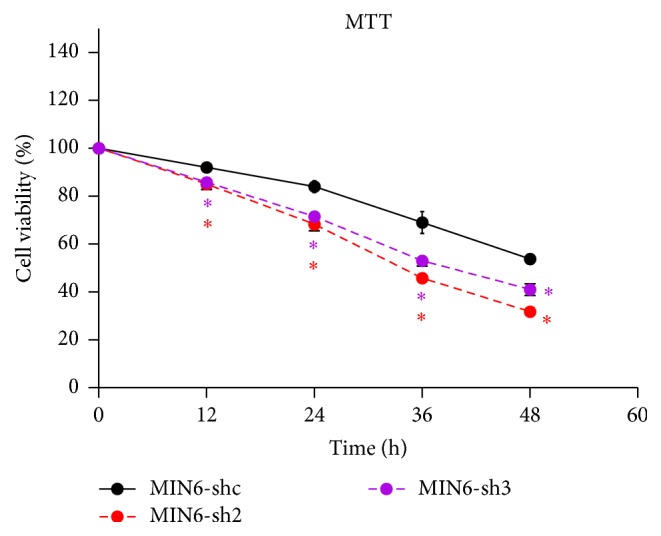
Nr2e1 knockdown MIN6 cells exhibit increased sensitivity to palmitate. Comparison of cell viability between control (MIN6-shc) and Nr2e1 silenced (MIN6-sh2 and MIN6-sh3) cells exposed to palmitate by MTT assay. MIN6-shc, MIN6-sh2, and MIN6-sh3 cells were cultured in complete medium with 0.5 mM palmitate for 48 h. Data represent means ± SD of four observations; ^*∗*^ *P* ≤ 0.05 (comparing silenced versus MIN6-shc cells).

To investigate the role of Nr2e1 in the survival of MIN6 cells, we investigated cell viability in MIN6 cells exposed to palmitate using MTT analysis. As shown in Figure 2, after 48 hours of palmitate exposure, MIN6-shc, MIN6-sh2, and MIN6-sh3 cells all exhibited decreased cell viability. Compared with the control cells, knockdown of Nr2e1 caused a more significant reduction in the percentage of viable cells.

### 3.3. Nr2e1 Silencing Suppressed Proliferation of MIN6 Cells

**Figure 3:**
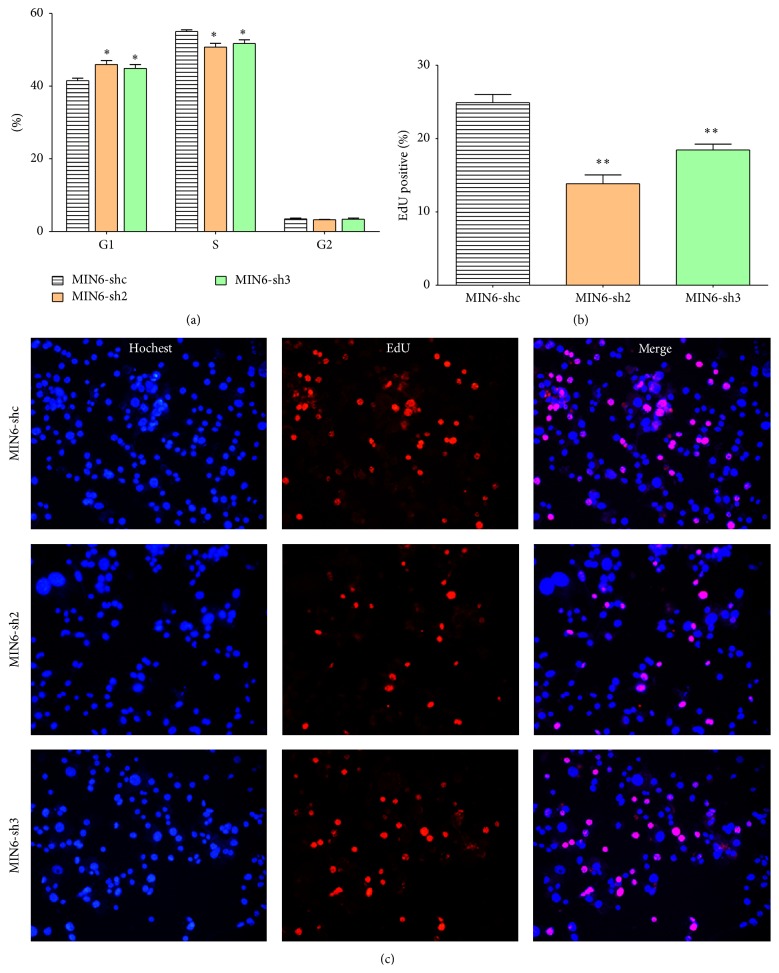
Nr2e1 silencing suppressed proliferation of MIN6 cells. (a) Comparison of cell-cycle distribution between control (MIN6-shc) and Nr2e1 silenced (MIN6-sh2 and MIN6-sh3) cells. (b) Percentage of EdU+ MIN6 cells cultured with EdU for 4 h. (c) Representative images (20x magnification) of MIN6 cells labeled with EdU. Data represent means ± SD of three observations; ^*∗*^ *P* ≤ 0.05; ^*∗∗*^ *P* ≤ 0.01 (comparing silenced versus MIN6-shc cells).

To determine if the decreased beta cell viability resulted from changes to beta cell proliferation, we investigated MIN6 cell proliferation rates by EdU incorporation assay. Labeling of cells with EdU and Hochest 33342 showed that, compared with the MIN6-shc cells, the knockdown of Nr2e1 decreased the percentage of EdU-positive cells (Figures 3(b) and 3(c)). The result suggests that Nr2e1 silencing could inhibit MIN6 cell proliferation. We then checked if the knockdown of Nr2e1 altered MIN6 cells cell-cycle distribution. The result showed that, compared to MIN6-shc cells, MIN6-sh2 cells and MIN6-sh3 cells had an increased percentage of G0/G1-phase cells and decreased percentage of S-phase cells (Figure 3(a)). These results indicate that MIN6-sh2 and MIN6-sh3 cells proliferate at a lower rate due to a partial inhibition of the G0/G1 phase of the cell cycle.

### 3.4. Nr2e1 Silencing Enhanced Apoptosis of MIN6 Cells

**Figure 4:**
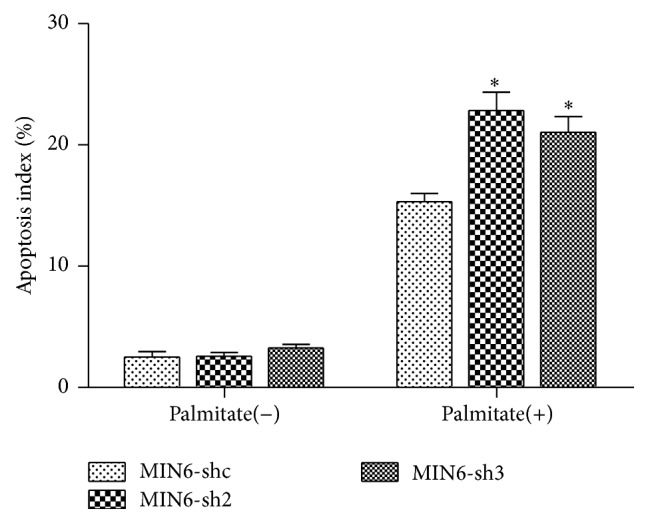
Nr2e1 silencing enhanced apoptosis of MIN6 cells. Comparison of cell apoptosis between control (MIN6-shc) and Nr2e1 silenced (MIN6-sh2 and MIN6-sh3) cells exposed to palmitate. MIN6-shc, MIN6-sh2, and MIN6-sh3 cells were cultured in complete medium with 0.5 mM palmitate overnight. Data represent means ± SD of three observations; ^*∗*^ *P* ≤ 0.05 (comparing silenced versus MIN6-shc cells exposed to palmitate).

We also studied effects of Nr2e1 silencing on MIN6 cells apoptosis in response to palmitate. At basal conditions, MIN6-shc, MIN6-sh2, and MIN6-sh3 cells displayed similar cell apoptosis rates. When stressed by overnight exposure to 0.5 mM palmitate, however, Nr2e1 silencing resulted in augmented cell apoptosis rates compared with control cells (Figure 4).

### 3.5. Nr2e1 Silencing Exaggerates Lipotoxicity-Induced Beta Cell Dysfunction

**Figure 5:**
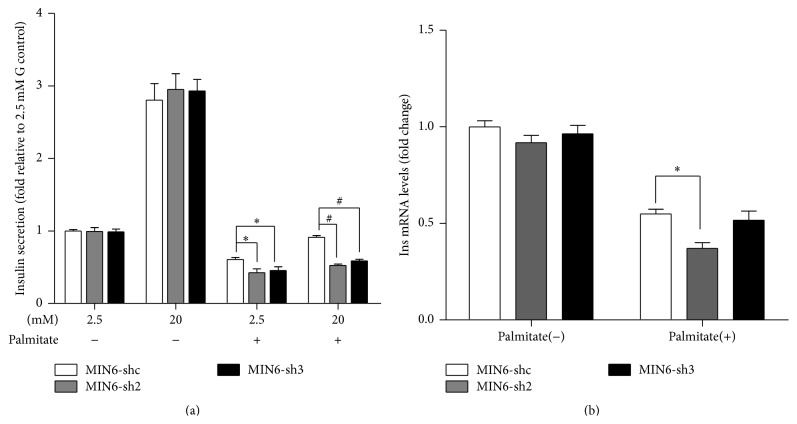
Nr2e1 silencing exaggerates lipotoxicity-induced beta cell dysfunction. (a) Insulin secretion in response to 2.5 and 20 mM glucose in MIN6-shc, MIN6-sh2, and MIN6-sh3 cells preincubated during 48 h with or without 0.5 mM palmitate. Results are means ± SD of 3 independent experiments. ^*∗*^ *P* < 0.05 compared to MIN6-shc in 2.5 mM glucose. ^#^ *P* < 0.05 compared to MIN6-shc in 20 mM glucose. (b) Insulin mRNA levels of MIN6-shc, MIN6-sh2, and MIN6-sh3 cells incubated during 48 h with or without 0.5 mM palmitate. Data represent means ± SD of three observations. ^*∗*^ *P* < 0.05 compared to MIN6-shc incubated with palmitate.

To explore the potential impact of Nr2e1 deficiency in beta cell function, GSIS was measured in MIN6-shc, MIN6-sh2, and MIN6-sh3 cells. In normal conditions, Nr2e1 downregulation in MIN6 cells did not affect the dose-response to glucose (Figure 5(a)). Also the insulin mRNA levels did not differ between control and Nr2e1 silenced cells (Figure 5(b)). So we next tested whether Nr2e1 alters GSIS in lipotoxic conditions. As expected, treatment with 0.5 mM palmitate for 48 h impaired GSIS and reduced mRNA level of insulin in MIN6 cells. These effects were more obvious in Nr2e1 silenced cells (Figures 5(a) and 5(b)).

### 3.6. Nr2e1 Silencing Augments Palmitate-Induced Oxidative Stress

**Figure 6:**
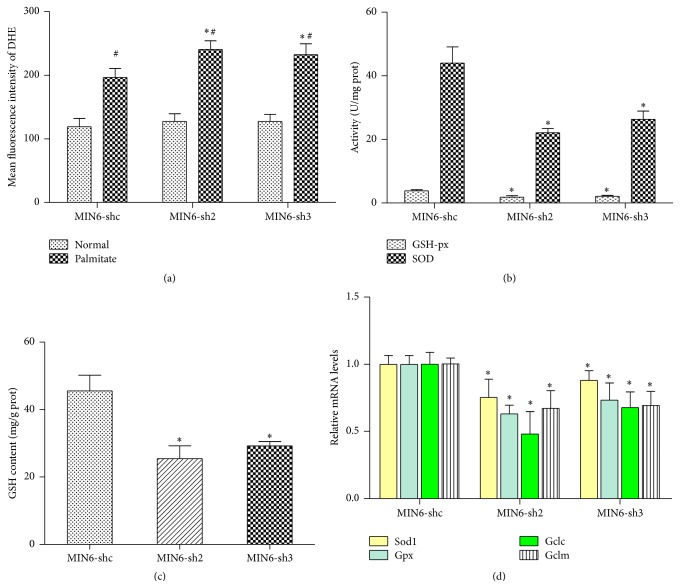
Nr2e1 silencing affects oxidative stress and antioxidant enzyme levels. (a) Comparison of intracellular levels of ROS between control (MIN6-shc) and Nr2e1 silenced (MIN6-sh2 and MIN6-sh3) cells. Cells were cultured in complete medium with or without 0.5 mM palmitate for 24 h and then the fluorescence intensity of DHE was measured using flow cytometry. Data represent means ± SD of three observations; ^#^ *P* ≤ 0.05 compared to cells in normal conditions. ^*∗*^ *P* ≤ 0.05 compared to MIN6-shc cells. (b) SOD and GSH-Px activity decreased in MIN6-sh2 and MIN6-sh3 cells. (c) GSH content decreased in MIN6-sh2 and MIN6-sh3 cells. (d) The relative mRNA expressions of Sod1, Gpx, Gclc, and Gclm were downregulated by Nr2e1 silencing. Data represent means ± SD of three observations; ^*∗*^ *P* ≤ 0.05; all comparisons with MIN6-shc cells.

Because Nr2e1 knockdown MIN6 cells exhibit increased sensitivity to lipotoxicity, we investigated the underlying mechanism that contributes to beta cell survival. It is well known that oxidative stress is a mechanism whereby lipotoxicity exerts harmful effects to damage beta cells, so we next investigated the levels of oxidative stress in MIN6-shc, MIN6-sh2, and MIN6-sh3 cells. Flow cytometry analysis using a ROS fluorescent probe (DHE) demonstrated an increase in ROS upon incubation of MIN6 cells with palmitate for 24 hours. Notably, this effect of palmitate was enhanced in MIN6-sh2 and MIN6-sh3 cells (Figure 6(a)). Because of low antioxidant enzyme production, beta cells have limited ability to counter ROS production. To determine if the promotion in oxidative stress in MIN6-sh2 and MIN6-sh3 cells resulted from decreases in antioxidant enzyme levels, we measure the activities of superoxide dismutase (SOD) and glutathione peroxidase (GSH-Px); results showed that Nr2e1 silencing inhibited the activity of GSH-Px and SOD activity and downregulated intracellular GSH content in MIN6 cells (Figures 6(b) and 6(c)). In addition, the mRNA expressions of Sod1, Gpx, and the GCL subunits Gclc and Gclm were measured. Significant changes were confirmed in the mRNA expressions of Sod1 and Gpx, which was perfectly consistent with that in their activities (Figure 6(d)). The levels of Gclc and Gclm mRNA were also substantially attenuated by Nr2e1 silencing (Figure 6(d)). These results indicate that Nr2e1 deficiency induced oxidative stress via ROS overproduction followed by disorder in the oxidant/antioxidant system.

### 3.7. Nr2e1 Silencing Suppressed Expression of Nrf2 in MIN6 Cells

**Figure 7:**
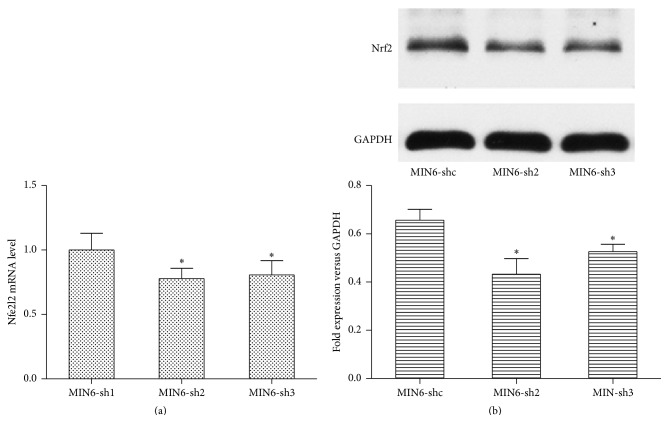
Effect of Nr2e1 on Nrf2 expression levels. (a) Relative expression level of Nfe2l2 mRNA in MIN6-shc, MIN6-sh2, and MIN6-sh3 cells. (b) Nrf2 protein levels. Results are means ± SD. ^*∗*^ *P* < 0.05 compared to MIN6-shc.

Because of the decreases observed in antioxidant enzymes levels, we hypothesized that Nr2e1 may modify key regulators of antioxidant genes. The Nrf2 transcription factor is an important activator of antioxidant genes. We next examined levels of Nrf2 protein and its corresponding mRNA (Nfe2l2). As shown in Figure 7, compared with control cells, knockdown of Nr2e1 resulted in a reduction in Nfe2l2 mRNA level. Immunoblots of MIN6 cells showed that Nrf2 protein levels paralleled the observed transcript levels.

## 4. Discussion

Previous studies have well described Nr2e1 activities in the nervous system, but only limited information is available about its function elsewhere. In the present study, we have demonstrated that Nr2e1 can control beta cells survival via regulating oxidative stress.

Free fatty acid-induced toxicity may recapitulate lipotoxicity seen in type 2 diabetes. Saturated fatty acids, including palmitic and stearic acids, were particularly cytotoxic to beta cells. Based on the facts that (I) lipotoxicity may impact beta cell mass by reducing beta cell proliferation [20], (II) after 48 hours of palmitate exposure, Nr2e1-silenced MIN6 cells displayed a decreased cell viability compared with the control cells, and (III) Nr2e1 is an essential gene for neural stem cell proliferation, we hypothesized that Nr2e1 may also promote or maintain proliferation in beta cells. Consistent with the hypothesis, our cell proliferation and cell-cycle analysis demonstrated that Nr2e1 silencing in MIN6 cells resulted in decreased proliferation and a partial G0/G1 cell-cycle arrest. In agreement with our results, others reported that Nr2e1 plays important roles in the proliferation of both embryonic and adult NSC, retinal progenitor cells, and prostate cancer cells [10, 16, 21–24]. Nr2e1 knockdown cells also displayed an enhanced apoptosis rate when exposed to palmitate. Increased apoptosis in the absence of Nr2e1 is indicative of synergistic roles in the cell survival pathway apart from the well established role in proliferation for which detailed studies are needed.

Previous study indicates that Nr2e1 is involved in regulation of intracellular calcium levels [11]. Cacna1d and Cacng5 are two L type, voltage-gated calcium channels and their expressions were notably upregulated in the Nr2e1−/− retinas. Plce1 is another gene regulating intracellular calcium level. Activation of Plce1 generates IP3 and DAG by PIP2. Increased levels of IP3 cause calcium release from intracellular stores via the IP3 receptors. Plce1 was identified as a direct downstream target of Nr2e1 transcriptional regulation [11]. It is well known that glucose-stimulated insulin secretion is associated with intracellular Ca^2+^. In view of this, we speculate that Nr2e1 may influence the insulin secretion. Interestingly, in normal conditions, we observed no differences in insulin secretion between control and Nr2e1-silenced MIN6 cells. However, when stressed by exposure to 0.5 mM palmitate, Nr2e1 silencing exaggerated palmitate-induced impairment in insulin secretion. It is likely that Nr2e1 is not required for the normal response to glucose. But, in lipotoxic condition, its deficiency impaired insulin secretion via augmenting oxidative stress. It is also likely that Nr2e1, rather than being an initiator, instead modulates insulin secretion synergistically with other factors. The impairment in insulin secretion caused by Nr2e1 deficiency is sufficiently compensated by other factors in normal condition but not in lipotoxic condition.

Increased metabolism of FFAs through mitochondrial oxidation will result in an increased mitochondrial membrane potential and superoxide production [25]. Increased superoxide production causes increased exposure of the cell to ROS. Here we show that palmitate can lead to increased beta cell ROS and this effect was notably enhanced by Nr2e1 silencing. All aerobic cells contain a battery of defenses against reactive oxygen species. The primary enzymatic defenses are SOD, which catalyses the conversion of superoxide radicals into H_2_O_2_ plus Cat and Gpx, both of which destroy H_2_O_2_. While it is important to realize that the production of ROS can have important regulatory functions on intermediary metabolism by altering the cellular redox state, the beta cell in which the expression levels of antioxidants enzymes are particularly low has limited defense against excess ROS production [26, 27]. In the current study, our studies provide important new insight, as we demonstrate that Nr2e1 silencing decreases the expression of antioxidant proteins, and therefore may impair the beta cell capacity to remediate oxidative stress.

Because of the decreases in Gpx and SOD1, we considered the possibility that Nr2e1 might affect the levels of Nrf2. Nrf2 is a powerful protein that upregulates antioxidant and phase II detoxification enzymes and protects cells from more serious oxidative damage for cell survival [28]. Once released it migrates into the cell nucleus and bonds to the DNA at the location of the antioxidant response element which is the master regulator of the total antioxidant system [29]. We show that Nrf2 levels are reduced by Nr2e1 silencing. Whether this effect on Nrf2 is a direct consequence of Nr2e1 effects on Nrf2 itself, or upon its regulatory proteins, is an ongoing area of investigation in our group. In addition, the decrease of Nrf2 level in MIN6-shNr2e1 cells was mild. So we speculate that, besides Nrf2, Nr2e1 may also regulate oxidative stress via other pathways which require further investigations.

Taken together, the current study provides evidence showing that nuclear orphan receptor Nr2e1 performs an essential role in regulating beta cell survival and function via regulation of oxidative stress. Whether or not Nr2e1 is involved in the etiology of beta cell failure and hyperglycemia in T2D rodent models will require further investigations.

## Supplementary Material

Primer sequences used for RT-PCR analyses in MIN6 cells.
