# Supplementary material for: Nr2e1 Deficiency Augments Palmitate-Induced Oxidative Stress in Beta Cells
Source: Oxid Med Cell Longev. 2015 Nov 16;2016:9648769. doi: 10.1155/2016/9648769 (PMC4663339; doi:10.1155/2016/9648769)
Supplement: Supplementary file 1 — Primer sequences used for RT-PCR analyses in MIN6 cells. [file 9648769.f1.docx]

**Supplemental Table 1. Primer sequences.**

| **Gene** | **Species** | **Forward primer** | **Reverse primer** |
| --- | --- | --- | --- |
| **ACTB** | **Mouse** | GTGACGTTGACATCCGTAAAGA | GTAACAGTCCGCCTAGAAGCAC |
| **Nr2e1** | **Mouse** | GGTTCAGACAGCTCCGATTAGAC | TGGAGAGCGGCAATGGCGGCAGC |
| **Sod1** | **Mouse** | GGCCCGGCGGATGA | CGTCCTTTCCAGCAGTCACA |
| **Gpx** | **Mouse** | ACAGTCCACCGTGTATGCCTTC | CTCTTCATTCTTGCCATTCTCCTG |
| **Gclc** | **Mouse** | TGGCCACTATCTGCCCAATT | GTCTGACACGTAGCCTCGGTAA |
| **Gclm** | **Mouse** | GCCACCAGATTTGACTGCCTTT | CAGGGATGCTTTCTTGAAGAGCTT |
| **Ins** | **Mouse** | AACCCACCCAGGCTTTTGTC | TCCAACGCCAAGGTCTGAAG |
| **Nrf2** | **Mouse** | CGAGATATACGCAGGAGAGGTAAGA | GCTCGACAATGTTCTCCAGCTT |
